# The Effect of Dissimilatory Manganese Reduction on Lactate Fermentation and Microbial Community Assembly

**DOI:** 10.3389/fmicb.2019.01007

**Published:** 2019-05-16

**Authors:** Breda Novotnik, Jackie Zorz, Steven Bryant, Marc Strous

**Affiliations:** ^1^Department of Geoscience, University of Calgary, Calgary, AB, Canada; ^2^Department of Chemical and Petroleum Engineering, University of Calgary, Calgary, AB, Canada

**Keywords:** manganese reduction, birnessite, fermentation, mixed culture, microbial community, *Shewanella*

## Abstract

Fermentation and dissimilatory manganese (Mn) reduction are inter-related metabolic processes that microbes can perform in anoxic environments. Fermentation is less energetically favorable and is often not considered to compete for organic carbon with dissimilatory metal reduction. Therefore, the aim of our study was to investigate the outcome of the competition for lactate between fermentation and Mn oxide (birnessite) reduction in a mixed microbial community. A birnessite reducing enrichment culture was obtained from activated sludge with lactate and birnessite as the substrates. This enrichment was further used to test how various birnessite activities (0, 10, 20, and 40 mM) affected the rates of fermentation and metal reduction, as well as community composition. Increased birnessite activity led to a decrease of lactate consumption rate. Acetate and propionate were the main products. With increasing birnessite activity, the propionate/acetate ratio decreased from 1.4 to 0.47. Significant CO_2_ production was detected only in the absence of birnessite. In its presence, CO_2_ concentrations remained close to the background since most of the CO_2_ produced in these experiments was recovered as MnCO_3_. The Mn reduction efficiency (Mn(II) produced divided by birnessite added) was the highest at 10 mM birnessite added, where about 50% of added birnessite was reduced to Mn(II), whereas at 20 and 40 mM approximately 21 and 16% was reduced. The decreased birnessite reduction efficiency at higher birnessite activities points to inhibition by terminal electron acceptors and/or its toxicity which was also indicated by retarded lactate oxidation and decreased concentrations of microbial metabolites. Birnessite activity strongly affected microbial community structure. Firmicutes and Bacteroidetes were the most abundant phyla at 0 mM of birnessite. Their abundance was inversely correlated with birnessite concentration. The relative sequence abundance of Proteobacteria correlated with birnessite concentrations. Most of the enriched populations were involved in lactate/acetate or amino acid fermentation and the only previously known metal reducing genus detected was related to *Shewanella* sp. The sequencing data confirmed that lactate consumption coupled to metal reduction was only one of the processes occurring and did not outcompete fermentation processes.

## Graphical Abstract

The co-occurrence of lactate fermentation and lactate oxidation with dissimilatory birnessite reduction by a mix microbial community with subsequent methanogenesis. When the ratio of propionate/acetate is equal or greater than 1.5 the dominant process of lactate consumption is fermentation. When the ratio is lower than 1.5 lactate is primarily consumed for birnessite reduction.

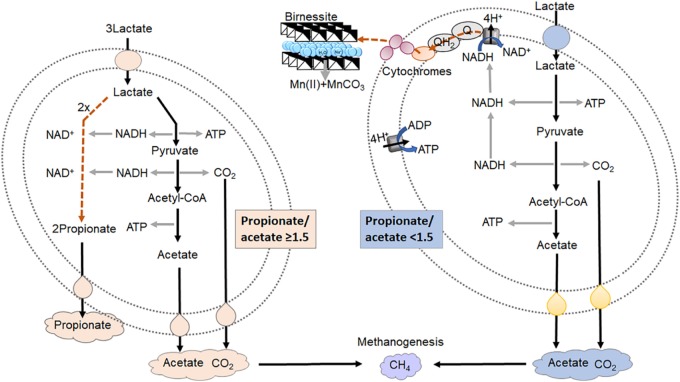

## Introduction

Microbial dissimilatory reduction of mineral oxides such as iron (Fe) and manganese (Mn) hydroxides influences carbon cycling as well as speciation of redox sensitive metals in the environment. It refers to a process where microbes reduce metals for respiration rather than assimilation. Aquatic and subsurface environments are rich in trivalent Fe [Fe(III)] and tri/tetravalent Mn [Mn(III/VI)] minerals that can support a variety of dissimilatory metal-reducing bacteria (DSMRB) when oxygen or other terminal electron acceptors are absent. Most microbes that can reduce Fe(III) can also reduce Mn(IV), and vice versa, and belong to a variety of different taxa, including *Firmicutes, Proteobacteria, Deferribacteres, Thermotogae*, and *Actinobacteria* (Lovley, 1991; Esther et al., 2014).

Amongst the microorganisms that can reduce metal oxides, there are organisms that do not conserve energy from this process but still perform this reaction as a sink for electrons. These include many fermentative and sulfate reducing Bacteria and Archaea (Lovley, 2013 and references therein). The most well-known microorganisms among those that do conserve energy for growth include *Shewanella* spp. (Hunt et al., 2010) and *Geobacter* spp. (Methé et al., 2003; Esther et al., 2014). The genera of *Shewanella* and *Geobacter* are the most studied metal-reducing microorganisms. They are not only capable of reducing insoluble Fe(III) and Mn(IV) oxides (Bretschger et al., 2007; Von Canstein et al., 2008; Kouzuma et al., 2012; Richter et al., 2012), but also soluble uranium(VI) (Cologgi et al., 2014) and chromium(VI) (Liu et al., 2002; Gong et al., 2018).

Manganese is a redox sensitive element and it undergoes many oxidation–reduction reactions in the natural environment. It occurs in oxidation states from 0 to +7, but only 2+, 3+, and 4+ have biological importance (Das et al., 2011). Manganese is readily reduced/oxidized by microbes, but it can also be abiotically reduced by Fe(II), sulfide, uraninite, and arsenite (Burdige and Nealson, 1985; Ying et al., 2011; Wang et al., 2013; Johnson et al., 2016) and oxidized by oxygen (Luther, 2007). In terms of microbial Mn(IV) reduction, the nature of the Mn(IV) oxide mineral has a major influence on the rate and extent of reduction. The most common Mn(IV) mineral studied is birnessite (Burdige et al., 1992; Horvath et al., 2014; Zhang et al., 2015), with the chemical formula Na_0.58_(Mn_1.42_^4+^,Mn_0.58_^3+^)O_4_×1.4H_2_O (Post et al., 2002; Fischer et al., 2018). Once birnessite is reduced, the Mn(II) ions are much more soluble than the mineral itself. Some Mn(II) ions will remain in aqueous solution, but the majority will still form a white precipitate of hodochrosite (MnCO_3_) (Chubar et al., 2015).

**FIGURE 1:**
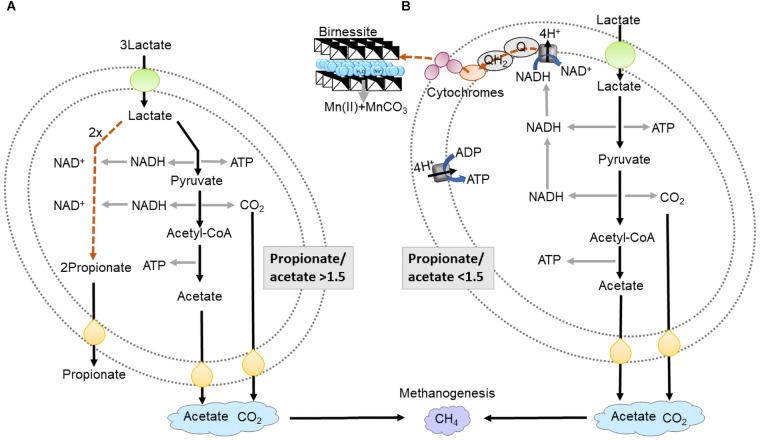
Schematic presentation of lactate fermentation **(A)** and dissimilatory birnessite reduction **(B)**. In lactate fermentation lactate is oxidized to pyruvate, generating ATP and NADH in the oxidative branch. Pyruvate is further oxidized to CO_2_ and acetate, giving yield to ATP and 2NADH. NADH is re-generated in the reductive branch forming propionate. In dissimilatory birnessite reduction lactate is oxidized to pyruvate then acetyl-CoA which is oxidized to acetate yielding ATP and 2NADH. NADH is regenerated by donating electrons to the NADH dehydrogenase then electrons are transferred via cytochromes extracellularly to birnessite. The energy yield per 1 mole of lactate is around 2/3 of ATP in fermentation and 6 ATP molecules in birnessite reduction.

Most Mn(III/IV) oxides are poorly soluble at near neutral pH. This creates a challenge for microorganisms, since the cell envelopes are impermeable to the minerals. Several mechanisms have evolved that help respire the minerals. First, electrons can be transferred from the inner membrane quinone/quinol pool through the periplasm to the outer membrane and onto the surface of the mineral (Shi et al., 2007) (Figure 1B). In *Shewanella* spp. the c-type cytochromes CymA, MtrA, MtrC, and OmcA along with a non-heme protein MtrB play an important role in this electron transfer. Second, a variety of microorganisms can produce siderophores that solubilize the metal ion from the oxide, so that it can be reduced inside the periplasm (Kouzuma et al., 2012). Third, some microorganisms synthesize low-molecular-weight organic molecules (“shuttles”) that transfer electrons between microorganisms and mineral electron acceptors (Von Canstein et al., 2008). Small sulfur molecules (Straub and Schink, 2004), dissolved as well as solid phase humic substances (Nevin and Lovley, 2000; Straub and Schink, 2004; Roden et al., 2010), and endogenous electron shuttles such as flavins (Marsili et al., 2008), which are produced by bacteria themselves, have also been observed/proposed to be involved in electron shuttling and dissimilatory metal reduction.

In anoxic environments and in the absence of terminal electron acceptors such as Mn(IV) oxides, microbes perform fermentation (Figure 1A). Fermentation and dissimilatory metal reduction, although both anaerobic processes, differ significantly in terms of redox reactions, ATP synthesis and electron transport (Figure 1). Fermentation processes are sometimes considered marginal, only occurring when electron acceptors have been fully consumed. From this perspective, fermentation would not be expected to directly compete for organic carbon with dissimilatory metal reduction, since the former is energetically far less favorable (Jin, 2012; Madigan et al., 2017). For fermentation of lactate, the substrate used in this study, one third of the lactate is oxidized to acetate and CO_2_ (Eq. 1) leading to production of 2 ATP and 2 NADH. To regenerate NAD^+^, two thirds of the lactate are reduced to propionate, without any ATP production (Figure 1A). In practice, previous research has shown that the ratio between propionate and acetate will vary, depending on the microorganisms executing the process, as well as the growth conditions (Seeliger et al., 2002).

(1) $$3{\mathrm{lactate}}^{-}\to 2{\mathrm{propionate}}^{-}+{\mathrm{lacetate}}^{-}+{\mathrm{HCO}}_{3}^{-}+H^{+}$$

Many fermentative bacteria have other options for disposal of NADH, for example the reduction of birnessite. By performing birnessite reduction lactate is oxidized to acetate and CO_2_ (Eq. 2) avoiding reduction to propionate and increasing ATP yield from 2/3 ATP per lactate to 2 ATP per lactate (Figure 1B).

(2) $$\mathrm{lactate}+2\mathrm{Mn}(\mathrm{IV})+2H_{2}O\to \mathrm{acetate}+2\mathrm{Mn}(\mathrm{II})+5H^{+}+{\mathrm{HCO}}_{3}^{-}$$

For comparison, complete oxidation of lactate to CO_2_, coupled to birnessite reduction, could yield 6–8 ATP per lactate, via the TCA cycle, proton translocation and ATP synthase (Figure 1B) (Parker et al., 2016). In dissimilatory metal reduction, electrons need to be transported extracellularly to the electron acceptor via a series of cytochromes (Figure 1B), which could kinetically bottleneck lactate metabolism. Therefore, tentatively the advantage of fermentation, although less energetically favorable, is that the microorganisms could convert the substrate faster, leading to higher substrate affinity. The aim of our study was to investigate the outcome of the competition for lactate between fermentation and birnessite reduction.

## Materials and Methods

### Preparation of Manganese Oxide

Manganese oxide was prepared as described before (McKenzie, 1971; Zhang et al., 2015). Briefly, 0.2 mol of KMnO_4_ (ACS reagent, Sigma-Aldrich, St. Louis, MO, United States) was weighted in 350 ml of ultrapure water and boiled. While boiling, 50 ml of concentrated HCl (Suprapur, Sigma-Aldrich, St. Louis, MO, United States) was added drop-wisely. After all the HCl was added, the solution was left to boil for an additional 30 min. After the solution cooled, the brown precipitate was harvested by centrifugation (Thermo Fischer Scientific, Waltham, MA, United States) (3500 rpm for 10 min) and the pellet washed with ultrapure water (18 MΩ, Milli-Q, Millipore, Burlington, MA, United States) until the pH of the solution was neutral. An additional step of washing was applied to remove any K^+^, Cl^-^ as well as aqueous Mn ions, by dialysing the oxide against ultrapure water using a 14 kDa molecular weight cut-off dialysis membrane (Spectrum Labs, Phoenix, AZ, United States). After the washing steps, manganese oxide was dried over night at 60°C. To assess particle size and morphology, transmission electron microscopy (TEM) (Tecnai F20 FEG-TEM, FEI Company, Hillsboro, OR, United States) analysis was performed at 200 kV. The particles were first sonicated in ethanol then casted onto a mesh TEM grid (C-Formvar 200, Ted Pella, United States) for imaging.

### Sequential Mn Extractions and ICP-MS Measurements

Manganese(II) was determined in two different fractions as described by Tessier et al. (1979) with slight modification. First an aliquot was withdrawn from the microcosms and centrifuged (14,000 rpm, 5 min). The supernatant was filtered and acidified with nitric acid (Suprapur, Sigma-Aldrich, St. Louis, MO, United States) to preserve the aqueous ions in the solution, representing the aqueous Mn fraction. The pellet was further extracted with 1 M sodium acetate (pH 5) for 24 h inside an anaerobic chamber to avoid Mn(II) oxidation and subsequent precipitation. After, samples were vortexed, centrifuged, and the supernatant was filtered, representing the carbonate bound Mn(II). All reagents used were ACS (American Chemical Society grade) reagents (Sigma-Aldrich, St. Louis, MO, United States). Dissolved manganese concentrations were determined using triple quadrupole ICP-MS (8900 Agilent, Santa Clara, CA, United States). To correct for signal drift during the measurements, germanium was used as an internal standard. The relative standard deviation of ICP-MS measurements was within 4%. The linear concentration range was between 0.1 and 1000 ng mL^-1^.

### HPLC Measurements

Lactate, acetate, formate, and propionate were analyzed using UltiMate 3000 HPLC system (Thermo Scientific, Waltham, MA, United States) equipped with an Aminex HPX-87H column (Bio-Rad, Hercules, CA, United States) and a UV detector. HPLC was operated under isocratic conditions with 5 mM H_2_SO_4_ (VWR, Radnor, PA, United States) as the mobile phase and a flow rate of 0.6 mL min^-1^ (UV detection at 210 nm). Column temperature was set to 60°C. The working concentration range was between 0.1 and 5 mM.

### GC Measurements

Carbon dioxide and CH_4_ measurements were performed simultaneously using 7890B GC system with flame ionization detector (FID) for CH_4_ and thermal conductivity detector (TCD) for CO_2_. The instrument operated under the following parameters: valve temperature: 125°C; oven temperature: 105°C; post-run at oven temperature of 50°C for 0 min. Sample loop: 0.25 mL. Column 1: gas He (105°C); Column 2: gas He 21 mL min^-1^ (at 105°C), constant flow; Column 3: gas He (105°C). TCD temperature: 200°C, reference flow: He 40 mL/min. FID: Heater T: 200°C, Air flow 400 ml/min, H_2_ fuel flow 50 ml/min. Column 1: 0.5 m × 1/8″ Hayesep N (80/100 mesh); Column 2: 6′ × 1/8″ Hayesep N (80/100 mesh); Column 3: 8′ × 1/8″ MS5A (60/80 mesh).

Dissolved CO_2_ and CH_4_ concentrations were calculated from partial pressures of corresponding gasses and Henry’s law constants of 29 atm M^-1^ for CO_2_ and 670 amt M^-1^ for CH_4_.

Reference gas mix from Praxair (Mississauga, ON, Canada) was used for calibration of the instrument. The working concentration range was between 0 and 20% of gas in the headspace.

### Enrichment of Manganese Reducing Microbial Consortia From Activated Sludge

To obtain a manganese oxide-reducing microbial consortium, returned activated sludge from a local waste water treatment plant (Bonnybrook Wastewater Treatment Plant, Calgary, AB, Canada), was used as the inoculum. Enrichments were set up in triplicates with lactate (40 mM) as the electron donor and manganese oxide (21.5 mM) as the electron acceptor. Although birnessite is an insoluble mineral, in this study we express birnessite activity in mM. This is done to enable assessment of the stoichiometry of the use of the electron acceptor and donor. Enrichment cultures and experiments were provided with 2.3–9.2 g L^-1^ of birnessite (10–40 mM). Ten ml of activated sludge was used as the inoculum. To ensure the pH was around neutral throughout the incubation, Coleville synthetic brine medium (CSBK) medium was used containing (per 1 L): 1.5 g NaCl, 0.05 g KH_2_PO_4_, 0.32 g NH_4_Cl, 0.22 g CaCl_2_, 0.54 g MgCl_2_ × 6H_2_O, and 0.1 g KCl. The medium was autoclaved, left to cool, then NaHCO_3_, autoclaved separately, was added to a final concentration of 1.14 g L^-1^. All chemicals were ACS reagents (Sigma-Aldrich, St. Louis, MO, United States). The final volume of the microcosms was 100 ml. The headspace of the microcosms was vacuumed and purged with N_2_ five times to ensure anaerobic conditions. The initial enrichments (transfer I) were incubated on a rotary shaker at 80 rpm, 30°C for 2 weeks. After that, 20 ml of the culture was transferred to fresh medium, containing lactate, manganese oxide, and fresh media as described before. These enrichments (transfer II) were incubated for 3 weeks, followed by another transfer (transfer III). At the final time point of incubation for the transfers II and III, samples were taken for DNA extraction. The culture obtained after transfer III was used in further experiments to test how varied manganese oxide activity affects fermentation, metal reduction, and community composition.

### Incubations of Enriched Microbial Consortia at Different Manganese Oxide Activity

Microcosms were prepared with the inoculum from the enriched birnessite-reducing culture (transfer III). All microcosms contained 40 mM lactate and were inoculated with 7 ml of the birnessite-reducing culture. CSBK medium was used to obtain a final volume of 35 ml. The manganese oxide activity was 0, 10, 20, or 40 mM. All experiments were done in triplicates. To control for any dissolution of birnessite and abiotic oxidation or possible adsorption of lactate by birnessite, abiotic controls were also set up with birnessite and lactate in CSBK media only, without any microorganisms.

Gas phase was measured at time 0, after 1, 2, 5, 10, 15, 20, and 25 days of incubation. Samples were withdrawn for organic acid and Mn analysis at days 0, 1, 2, 5, 15, 20, and 25.

After 25 days of incubation samples were also withdrawn for DNA extraction and 16S rRNA gene sequencing.

### 16S rRNA Gene Amplicon Sequencing

DNA was extracted from the initial activated sludge as well as from enrichment cultures at the final incubation time point using FastDNA Extraction Kit for Soil (MP Biomedicals, Santa Ana, CA, United States) according to the instruction manual. DNA was quantified using Qubit^TM^ dsDNA HS Assay Kit and Qubit 2.0 fluorometer (Thermo Fischer Scientific, Waltham, MA, United States).

A two step PCR method was used for Illumina 16S rRNA gene library preparation. First, the 16S rRNA genes were amplified from the DNA extracts using primers 515F-806R, that were designed to amplify prokaryotes (Bacteria and Archaea), targeting the V4 region of the 16S rRNA gene (Caporaso et al., 2010; Apprill et al., 2015; Parada et al., 2016). Primers were modified with Illumina MiSeq overhang adapters (Sharp et al., 2017). PCR mixtures contained 0.1 μM of the forward primer, 0.1 μM of the reverse primer (Integrated DNA Technologies, Inc., Coralville, IA, United States), 12.5 μL of 2× KAPA HiFi Hot Start Ready Mix (Kapa Biosystems, Wilmington, MA, United States), 0.5 mg mL^-1^ BSA (UltraPure, Thermo Fischer Scientific, Waltham, MA, United States) and 5 μL of template DNA. PCR reaction conditions were as follows: initial denaturation step 94°C for 3 min, followed by 35 cycles of 45 s at 94°C, 60 s at 50°C and 60 s at 72°C, and a 10 min final elongation at 72°C. All PCR reactions were performed in triplicates and pooled for further purification with AMPure XP (Beckman Coulter, Indianapolis, IN, United States) magnetic beads as per the manufacturer’s instructions. After, a second PCR was used to attach dual indices and Illumina sequencing adapters to the amplicons from the first PCR reaction, described in details by Sharp et al. (2017). After the second PCR reaction, products were purified using AMPure XP magnetic beads as per the manufacturer’s instructions. The concentration of dsDNA was determined again, then samples were pooled and normalized to obtain a final DNA concentration in the range 0.1 to 10 ng μg^-1^. Sequencing was performed using Illumina’s v3, 600-cycle (paired-end) reagent kit on a MiSeq benchtop sequencer (Illumina, Inc., San Diego, CA, United States).

### Amplicon Sequence Processing and Analyses

Paired-end raw reads obtained from Illumina were processed using the MetaAmp online pipeline (Dong et al., 2017). The input files of raw paired-end sequences were in FASTQ format. The MetaAmp workflow is described in details elsewhere (Dong et al., 2017). Briefly, the reads were first merged, generating a longer single read with corresponding quality control score. In our analysis a minimum length of overlap of 80 base pairs (bp) was specified for the merging options, with the maximum number of 8 bp mismatches. In the second stage, forward and reverse primers were trimmed. The primer-trimmed reads were subjected to quality filtering. For the quality filtering options, maximum number of differences to the primer sequence as well as maximum number of expected errors of 1 were allowed. The quality filtering and truncating were done using “usearch -fastq_filter” command with “-fastq_trunclen -fastq_maxee -fastaout” options in USEARCH. The remaining reads were truncated to a length of 250 bp and reads which had a higher number of total expected errors for the truncated read length were discarded. The reads with lengths shorter than the truncation length were also excluded from further analysis. In the third stage de-replication and removal of single reads and chimeric sequencing was performed, the high-quality reads were clustered into operational taxonomic units (OTUs) based on the 97% sequence identity threshold over 250 bases. All samples were rarefied to the number of reads in the smallest sample to avoid biases from different sampling depths. After rarefication, community similarities were measured using the Bray–Curtis index and alpha-diversity metrics (observed OTU richness, Inverse Simpson’s and Shannon diversity index) were calculated from the rarefied libraries using Mothur version 1.35.1. To test for community dissimilarities ANOSIM in Vegan R package (R Studio, 2015) was performed.

Amplicon sequences generated in this study are available through the NCBI Sequence Read Archive (BioProject Accession No. PRJNA528502).

## Results

### Enrichment of Birnessite-Reducing Microorganisms From Activated Sludge

**FIGURE 2:**
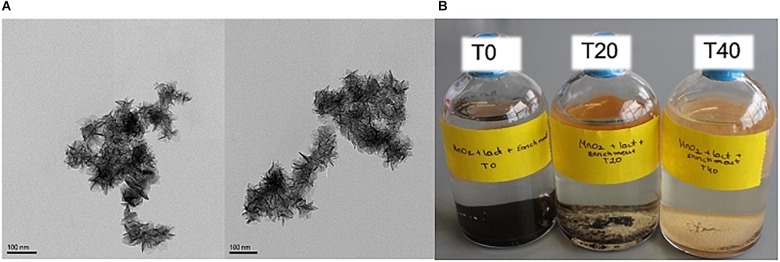
TEM microscopy image of birnessite **(A)**. Microcosms of enriched culture with birnessite and lactate at time 0 (T0), after 20 days (T20), and after 40 days (T40) of incubation **(B)**. At T0 only brown birnessite precipitate was noticed, as incubation time increased, birnessite was consumed and more biomass was visible, and the brown precipitate receded.

Activated sludge was used as the inoculum for enrichment, because of its high biological and functional diversity (Zhang et al., 2015), and tolerance to high dissolved metal concentrations (Novotnik et al., 2014). The Mn(IV) oxide used in our study was birnessite. Transmission electron microscopy visualized its typical “nanoflower”-like morphology, as shown in Figure 2A. Birnessite was used as the only electron acceptor (4.8 g L^-1^ equivalent to a birnessite activity of 21.5 mM) during enrichment, and lactate (40 mM) was supplied as the only electron donor. The enrichments were left until, after ∼40 days, clearing/consumption of the brown birnessite was observed, as shown in Figure 2B. Then, the culture was transferred into a fresh medium with the same initial birnessite activity and lactate concentration. The enriched culture attained after three transfers was then used for further experiments.

**FIGURE 3:**
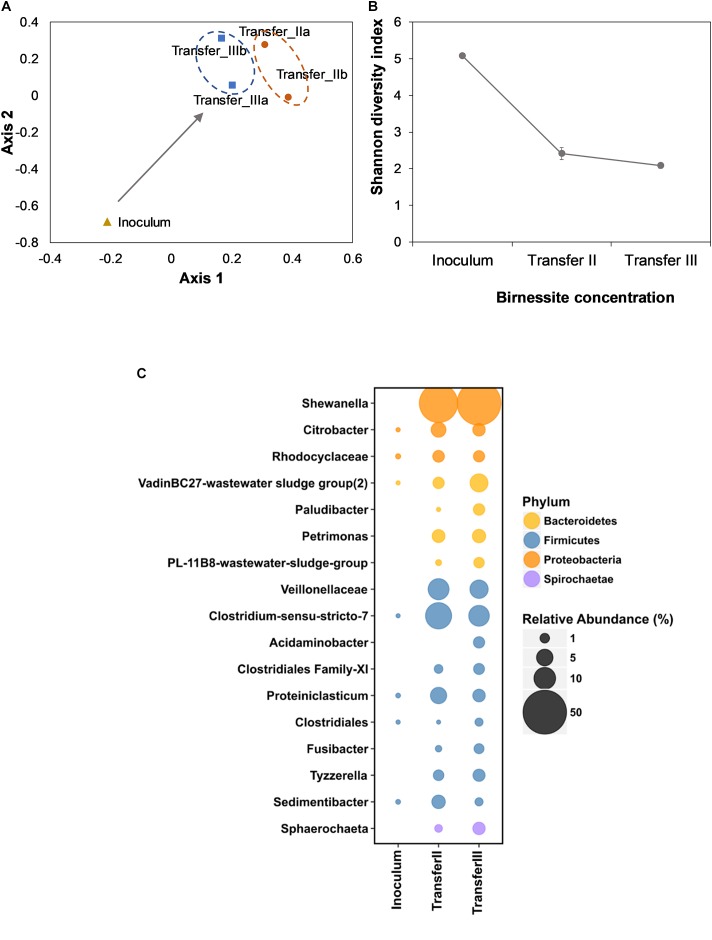
Non-metric multidimensional scaling (NMDS) analysis of the inoculum and after II and III transfer **(A)**. The duplicates for the community composition after II and III transfer were visualized individually instead of average. The Stress value for this system was 0.2. Shannon diversity index (SDI) **(B)** of the inoculum and of transfer II and III. Error bars indicate the standard deviation of duplicate experiments. The bubble chart **(C)** represents the relative abundance of 16S rRNA gene (%) in the inoculum and in the enrichment after II and III transfer at the Family or Genus level when classified. Size of the bubble relates to the relative abundance of 16S rRNA gene (%). For visualization purposes only OTUs with an abundance > 1% in either transfer II or III are shown. Both Proteobacteria and Firmicutes were enriched, with *Shewanella* being the most abundant OTU in birnessite enrichments. Average values of duplicate experiments were presented.

To assess the microbial community assembly during enrichment, 16S rRNA gene amplicons were sequenced. To visualize dissimilarities between the inoculum and the enrichment cultures, non-metric multidimensional scaling (NMDS) was employed. Figure 3A shows how the community composition changed from the inoculum to the community assembled after two and three transfers. The changes in the community were observed as the spatial separation between inoculum, transfer II and transfer III. The proximity of transfers II and III indicated that the enrichment was already almost complete after transfer II. The loss of diversity during enrichment was confirmed by decreased Shannon diversity index (Figure 3B).

In the initial activated sludge inoculum, a total of 1,050 OTUs were detected. After three transfers, only 158 OTUs remained. After the third transfer, Proteobacteria had the highest relative sequence abundance (51%), followed by Firmicutes (32%) and Bacteroidetes (12%). A population affiliated with *Shewanella* achieved the highest relative sequence abundance (55%, Figure 3C), despite its abundance being below the limit of detection in the inoculum. Other abundant proteobacteria were affiliated with *Citrobacter* (2.4%) and *Rhodocyclaceae* (1.8%). Among Bacteroidetes, the VadinBC27-wastewater sludge group (2.9%), *Paludibacter* (1.8%), *Petrimonas* (2.9%), and PL-11B8-wastewater sludge group (1.4%) were represented. Among Firmicutes, populations affiliated with *Veillonellaceae* (6.5%), *Clostridium-sensu stricto* (8.9%), *Acidaminobacter* (1.7%), *Clostridiales* Family XI incertae sedis (1.5%), *Proteiniclasticum* (2.5%), *Clostridiales* (0.5%), *Fusibacter* (1.2%), *Tyzzerella* (3.4%), and *Sedimentibacter* (0.5%) were enriched.

### The Effect of Birnessite Activity on Lactate Consumption

**FIGURE 4:**
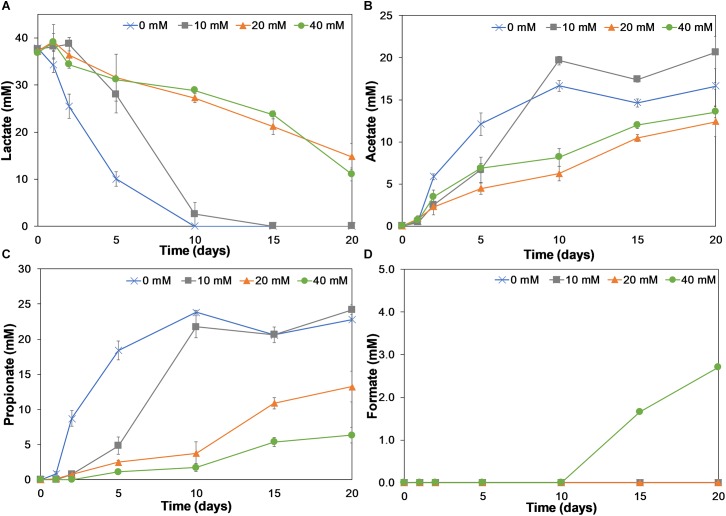
Lactate consumption **(A)** and acetate **(B)**, propionate **(C)**, and formate **(D)** production on incubation days 0, 1, 2, 5, 10, 15, and 20 for birnessite activities of 0, 10, 20, and 40 mM. Lactate consumption as well as acetate, propionate, and formate production were affected by birnessite presence. Error bars indicate the standard deviation of triplicate experiments.

To investigate how birnessite activity impacted lactate fermentation and community structure, four different initial birnessite activities were tested in triplicated separate microcosms with birnessite activities of 0, 10, 20, and 40 mM. The initial lactate concentration was 40 mM in all experiments. Figure 4A shows the consumption rate of lactate at different initial birnessite activities.

In the absence of birnessite, the consumption of lactate was faster than in its presence. At 0 mM of birnessite all lactate was consumed after 10 days of incubation, whereas at 10, 20, and 40 mM of birnessite, lactate was still detected at concentrations of 2.6, 27.2, and 28.8 mM, respectively. At the 20-day mark, lactate could not be detected in the 10 mM birnessite microcosms but was detected in the microcosms with 20 and 40 mM of birnessite.

In Figure 4B–D, the microbial metabolic products acetate, propionate, and formate, are presented. Acetate and propionate were produced in all microcosms, whereas formate was detected only at the end of the 40 mM birnessite incubation. For the 0 and 10 mM birnessite microcosms, lactate was completely fermented to acetate and propionate during the first 10 days. In 20 and 40 mM birnessite microcosms, lactate conversion was much slower. Even after 20 days, it was not completely converted. Acetate production in 20 and 40 mM birnessite microcosms reached only around 55% of the acetate produced in 10 mM birnessite microcosm. For propionate production, the effect of birnessite activity became even more apparent, with 75% less propionate produced in the 40 mM birnessite microcosms, compared to 10 mM experiments.

Table 1 shows the ratio between propionate and acetate production. The propionate to acetate ratio was around 1.5:1 throughout the 20-day incubation in the absence of birnessite. With increasing birnessite activities, less and less propionate was produced compared to acetate. The propionate/acetate ratio after 20 days decreased from 1.4 at 0 mM to 0.47 for the 40 mM birnessite microcosms.

**Table 1:** Ratio of propionate to acetate during 20-day incubation at 0, 10, 20, and 40 mM of birnessite.

Propionate/acetate						
	**Incubation days**					
	
**Birnessite**	**1**	**2**	**5**	**10**	**15**	**20**

0 mM	1.49	1.49	1.52	1.43	1.41	1.37
10 mM	0.00	0.31	0.73	1.11	1.19	1.17
20 mM	0.19	0.32	0.56	0.60	1.04	1.07
40 mM	0.00	0.00	0.16	0.21	0.45	0.47


In abiotic controls, the lactate concentrations remained stable throughout the incubation period, except for the final time point (20 days) in the 40 mM birnessite microcosms, where a ∼ 10% decrease of lactate was detected. In these experiments neither acetate, propionate, nor formate were detected.

**FIGURE 5:**
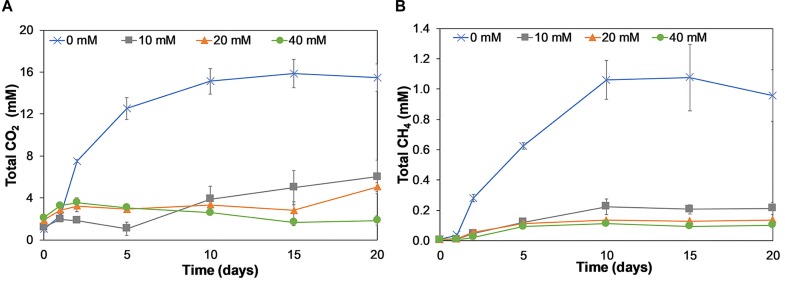
Production of dissolved and gaseous CO_2_ (mM) **(A)** and CH_4_ (mM) **(B)** on incubation days 0, 1, 2, 5, 10, 15, and 20 for birnessite activities of 0, 10, 20, and 40 mM. Both CO_2_ and CH_4_ were present at 0 mM birnessite and decreased with birnessite activities. Error bars represent the standard deviation of triplicate experiments.

As CO_2_ was also one of the expected end-products, we quantified the amounts of CO_2_ produced during lactate fermentation (Figure 5A).

Significant CO_2_ production (up to 15.5 mM) was detected in the absence of birnessite. In the presence of birnessite, CO_2_ concentrations remained close to the background and control experiments. In birnessite microcosms, we would expect more CO_2_ production, not less (Eq. 2), but most of the CO_2_ produced in these experiments was recovered as MnCO_3_ (see below). Although methane (CH_4_) is not a by-product of lactate oxidation, it could be produced by methanogenic microorganisms via hydrogenotrophic or acetoclastic methanogenesis in our microcosm incubations. The largest increase in CH_4_ concentration was recorded in 0 mM birnessite microcosms. Methane was also detected in the 10, 20, and 40 mM birnessite microcosms but the amount of CH_4_ detected declined with increasing birnessite activities. In the abiotic controls, no CH_4_ was detected.

### Birnessite Reduction and Dissolution of Aqueous Mn(II) and Precipitated Mn(II)-CO_3_

**FIGURE 6:**
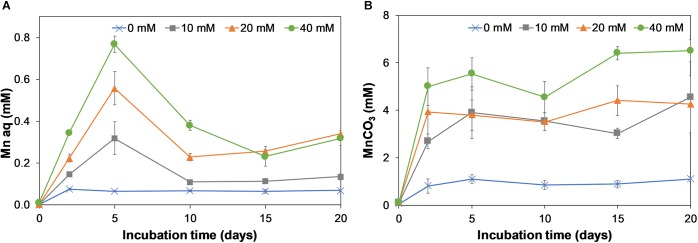
Production of aqueous Mn **(A)** and carbonate bound Mn **(B)** on incubation days 0, 2, 5, 10, 15, and 20 for 0, 10, 20, and 40 mM. Error bars represent the standard deviation of triplicate experiments.

Birnessite is a poorly soluble mineral and during its microbial reduction, aqueous Mn(II) ions are produced. These have a tendency to bind to carbonates, forming Mn(II)CO_3_ precipitates (Johnson et al., 2016). Therefore, we report aqueous Mn as well as Mn bound as carbonates in Figure 6A,B, respectively. For the aqueous Mn, an initial increase during the first 5 days of incubation was observed, followed by a drop at day 10. During the rest of incubation the Mn concentrations slowly increased. The decrease at day 10 might be due to precipitation of aqueous Mn as carbonate or phosphate bound Mn. Most reduced Mn was recovered in the form of MnCO_3_ (Figure 6B), which increased rapidly in the first 2 days of the experiment, and more gradually afterward.

The rate of formation of MnCO_3_ increased with birnessite activity. The formation of an insoluble reduced manganese carbonate precipitate could explain why little release of dissolved CO_2_ was observed in birnessite microcosms (Figure 5).

### Carbon and Electron Balance During Lactate Fermentation and Metal Reduction

The carbon balance was calculated by comparing the amount of carbon consumed, in the form of lactate, with the amount of carbon produced as products (acetate, propionate, formate, carbon dioxide, MnCO_3_, and methane) on the final day of the incubations (day 20). The number of carbons in lactate and each of its metabolic products was multiplied with the corresponding concentration change, leading to the values presented in Table 2. The lactate decrease in abiotic controls was also considered and was significant at 40 mM of birnessite. The “ΔC” column presents the balance of all carbon consumed and produced.

**Table 2:** Carbon balances of experiments with different amounts of birnessite added.

Birnessite added (mM)	Lactate-C	Acetate-C	Propionate-C	Formate-C	CO_2_-C	MnCO_3_-C	CH_4_-C	Abiotic C loss	Total C produced	ΔC
0	-113.4	+33.2	+68.3	0.0	+15.5	+1.2	+1.0	0.0	+119.2	**+5.8**
10	-117	+41.2	+72.6	0.0	+6.0	+4.9	+0.2	0.0	+124.9	**+7.9**
20	-67.3	+24.8	+39.7	0.0	+5.0	+4.6	+0.1	0.0	+74.3	**+7.0**
40	-77.2	+27.1	+19.0	+2.7	+1.9	+7.5	+0.1	12.6	+70.4	**-6.8**
**# of carbons**	**3**	**2**	**3**	**1**	**1**	**1**	**1**	**3**		


*Concentration changes (carbon-mM) associated with consumption of lactate (shown as negative values) and production of acetate, propionate, formate, CO_*2*_, MnCO_*3*_, and CH_*4*_ at the final day of incubation. Abiotic lactate-C decrease is also included and was only detected at 40 mM of birnessite. The “ΔC” column shows the difference between all carbons consumed and produced, with a positive value indicating net carbon production.*

For the experiments with 0, 10, and 20 mM of birnessite, more carbon was recovered than introduced with an error of 5–8 mM carbon (Table 2). For 40 mM of birnessite experiments not all carbon was recovered in the form of metabolites, with 7 mM of carbon missing. This inaccuracy is still within the margin of error. The detection of inorganic carbon, in the form of manganese bicarbonate and carbon dioxide, had the highest margin of error. Thus, most likely, dissimilatory metal reduction rates at 40 mM of birnessite might have been slightly higher. The electron balance table (Table 3) shows the balance between the degree of reduction of substrates and products. The electron balance was calculated for the final time point of the incubations. The electron balance was slightly negative at 0, 20, and 40 mM birnessite and slightly positive at 10 mM birnessite. For the 0 and 20 mM experiments, carbon (Table 2) was overestimated, hence together with a negative electron balance, it appeared that the concentration of an oxidized product (e.g., CO_2_) was overestimated. For the 40 mM birnessite microcosms, there was carbon missing in the products. Together with a negative electron balance, this indicated that the concentration of a reduced product (e.g., propionate) may have been underestimated. For the 10 mM birnessite microcosms, the combination of a positive electron balance with an overestimation of produced carbon pointed at the overestimation of the concentration of a reduced product (e.g., propionate).

**Table 3:** Electron balance of experiments with different amounts of birnessite added.

Birnessite added (mM)	Lactate	MnIV (MnO_2_)	Acetate	Propionate	Formate	MnII (as CO_3_)	MnII (aq)	Total CO_2_	CO_3_(^2-^) (from MnCO_3_)	total CH_4_	Electron balance
0	0	4.7	0	45.6	0	-2.2	-0.1	-61.8	-4.8	3.8	-**14.9**
10	0	18.7	0	48.4	0	-9.1	-0.3	-24.0	-19.7	0.9	**14.8**
20	0	18.4	0	26.5	0	-8.5	-0.7	-20.2	-18.4	0.6	-**2.4**
40	0	27.3	0	12.7	-5.4	-13.0	-0.6	-7.4	-28.2	0.4	-**14.3**
**Degree of reduction**	**0**	**-4**	**0**	**+2**	**-2**	**-2**	**-2**	**-4**	**-4**	**+4**	


*Electron balance was calculated for the final time point of incubation by using the “Degree of reduction” specified in the table. The column “Electron balance” indicates the net balance of reduced and oxidized products determined. The row “Degree of reduction” states the values used for each species.*

### The Effect of Birnessite on the Microbial Community Structure

**FIGURE 7:**
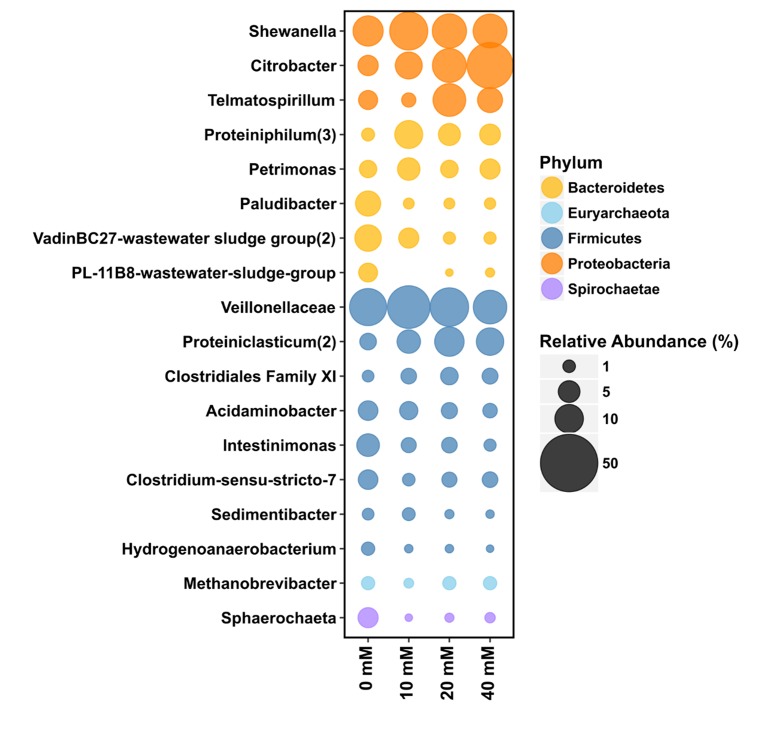
The bubble plots represent the relative abundance of 16S rRNA gene (%) in microcosms with 0, 10, 20, and 40 mM of birnessite at the Family or Genus level (when classified). The relative sequence abundance of Firmicutes and Bacteroidetes decreased with increasing birnessite activities whereas relative sequence abundance of Proteobacteria increased. Size of the bubble relates to the relative abundance of 16S rRNA gene (%). Average values of triplicate experiments were presented.

**FIGURE 8:**
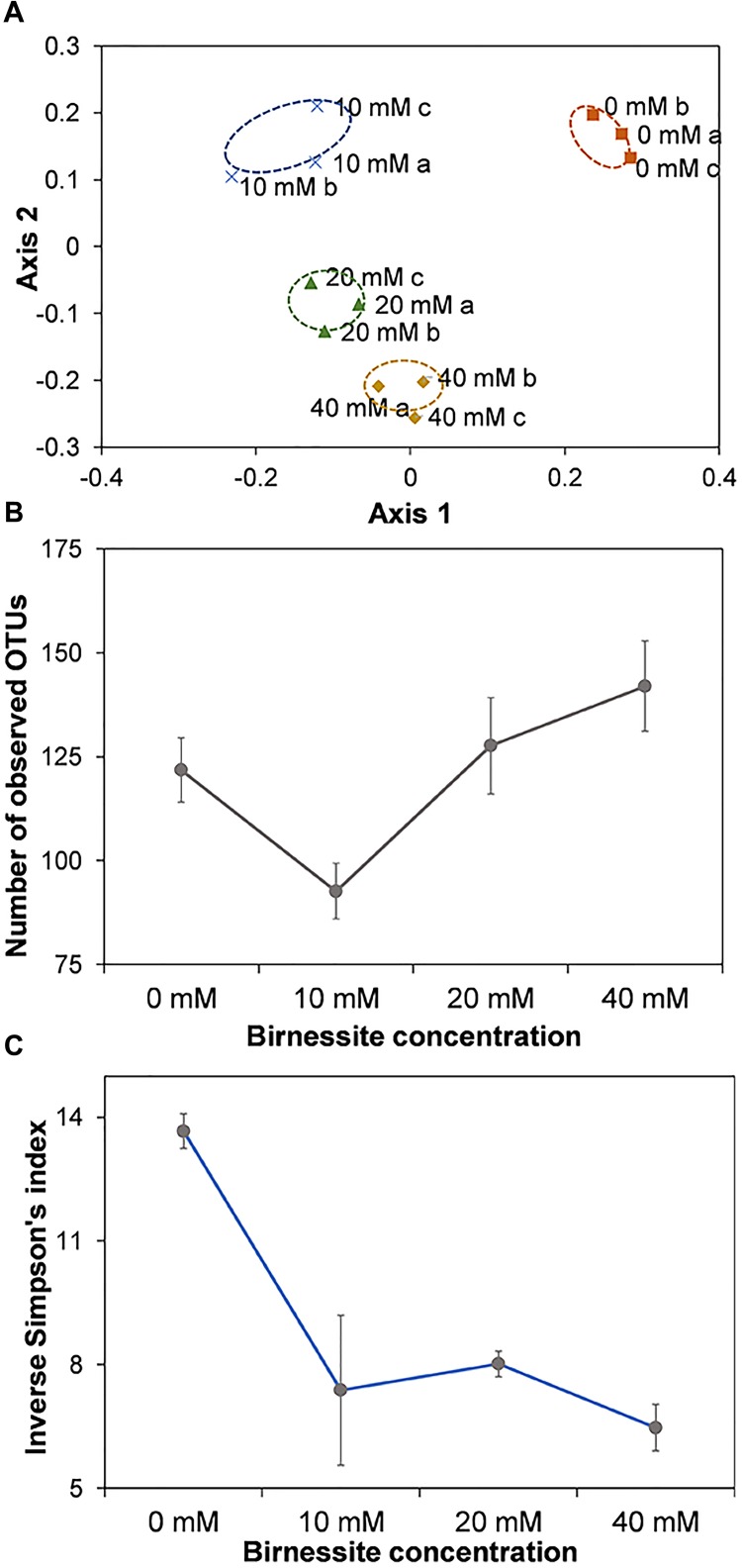
Non-metric NMDS of community compositions based on the Bray–Curtis dissimilarity distance **(A)**. The triplicates for each microcosm were visualized individually. The Stress value for this system is 0.11. Number of observed OTUs **(B)** and Inverse Simpson’s diversity index **(C)** at birnessite activities of 0, 10, 20, and 40 mM. Number of observed OTUs was the highest at 40 mM birnessite, but the Inverse Simpson’s index decreased with increasing birnessite activities. Error bars represent the standard deviation from average of the triplicates.

Birnessite activity strongly affected microbial community structure (Figure 7, 8). Firmicutes (47% relative sequence abundance) and Bacteroidetes (24%) were the most abundant phyla at 0 mM of birnessite. Their abundance decreased with increasing birnessite activity, to 32 and 11% at 40 mM birnessite, respectively. The relative sequence abundance of Proteobacteria increased with increasing birnessite concentrations, from 21% at 0 mM to 54% at 40 mM of birnessite. Figure 7 presents the microbial community composition, based on amplicon sequencing. The relative sequence abundances presented are averages of biological triplicates.

Among Proteobacteria, *Shewanella, Citrobacter*, and *Telmatospirillum* were the major genera present. For each of these taxa, their relative sequence abundance increased with increasing birnessite activity.

Among Bacteroidetes, the relative sequence abundance of the proteolytic genus *Proteiniphylum* increased with birnessite activity, whereas abundance of *Petrimonas, Paludibacter* and a representative of the VadinBC27-wastewater-sludge-group decreased with increasing birnessite activity.

Among Firmicutes the most abundant OTU was affiliated with *Veillonelaceae* and its relative abundance was the highest at low birnessite activity. The abundance of most other detected Firmicutes, for example those affiliated with *Acidaminobacter, Intestinimonas, Sedimentibacter*, and *Hydrogenoanaerobacterium* also decreased with birnessite activity. On the other hand, birnessite activity favored growth of a population affiliated with *Proteiniclasticum* (part of *Clostridiaceae*).

Methanogenic archaea affiliated with *Methanobrevibacter* were present in all microcosms, at around 1.2% relative sequence abundance, and were likely responsible for CH_4_ production, via hydrogenotrophic methanogenesis.

Community dissimilarities were visualized using NMDS. As can be seen from Figure 8A, the communities of each triplicate experiment clustered together, while there was clear spatial separation among communities with different birnessite activities. The Stress value for the system was 0.11 indicating that the NMDS represents the observed differences between communities in the reduced dimensions well. Analysis of similarities (ANOSIM) indicated that the dissimilarity between groups was statistically significant (*R*: 0.98, *p* < 0.001). The richness of the microbial communities, expressed as the number of observed OTUs (Figure 8B), varied between samples with different birnessite activities and was the lowest at 10 mM and highest at 40 mM. On the other hand, the Inverse Simpson’s diversity index, which considers the number of OTUs and also their abundance (Figure 8C), was highest at 0 mM and decreased with increased birnessite activities. Thus, not only the presence but also the amount of birnessite appeared to be a key factor influencing microbial diversity and composition.

## Discussion

Fermentative microorganisms and dissimilatory metal reducing bacteria utilize lactate and conserve energy in two very distinct ways. When lactate is fermented (Figure 1A), it is first oxidized to pyruvate, yielding ATP and NADH. Lactate or pyruvate are further reduced to propionate in the reductive branch, consuming NADH. In the oxidative branch pyruvate is oxidized to acetate and CO_2_, yielding ATP and NADH (Müller, 2001). In dissimilatory metal reduction (Figure 1B) lactate is oxidized to acetate and CO_2_, there is no formation of propionate since the electrons are transferred via transmembrane cytochrome system extracellularly to manganese oxides. ATP is generated via electron transport and chemiosmosis (Parker et al., 2016).

Our results indicated that with increased birnessite activities, the consumption rate of lactate was reduced. At the same time, a shift was observed from lactate fermentation to birnessite respiration of lactate, the latter proceeding at a slower rate. The presence of birnessite must have suppressed the activity of lactate fermenting bacteria, because otherwise, lactate oxidizing microbes (e.g., *Shewanella*) would not have been able to compete with the faster fermentative bacteria (Parker et al., 2016). The slower lactate consumption at higher birnessite activities was also reflected in acetate and propionate production which was far lesser in the microcosms with 20 and 40 mM of birnessite. Additionally, the propionate/acetate ratio decreased from a value of 1.4 at 0 mM, which is a typical ratio during fermentation, to 0.47 for the 40 mM birnessite incubations. As shown in Figure 1B and Eq. 2 (Lovley, 1991), when lactate fermentation is combined with Mn reduction, acetate is the main metabolic product. Hence, the increased acetate to propionate ratio at higher birnessite activity suggested that in those microcosms, species performing partial lactate oxidation/birnessite reduction outcompeted strictly fermentative species.

Carbon dioxide was highest in microcosms without any birnessite addition and with increasing birnessite activities its concentration decreased. This was due to delayed lactate consumption at increased birnessite activities but also due to increased Mn(II) production. Namely, at higher Mn(II) concentrations more MnCO_3_ was formed which decreased the dissolved carbonate concentrations in microcosms with birnessite present. Methane was primarily produced at 0 mM of birnessite. In these microcosms both *Hydrogenoanaerobacterium* and *Methanobrevibacter* were detected. These two bacterial populations may have co-existed in a syntrophic relationship. *Hydrogenoanaerobacterium* could provide substrates (acetate, CO_2_, and hydrogen) for methane formation by *Methanobrevibacter.* It appeared that the presence of birnessite suppressed methanogenesis, which might have been caused by toxicity of birnessite to methanogens, or to their syntrophic partners.

The Mn reduction efficiency [Mn(II) produced divided by birnessite added] was the highest at 10 mM birnessite added, where about 50% of added birnessite was reduced to Mn(II), whereas at 20 and 40 mM approximately 21 and 16% was reduced. The decreased birnessite reduction efficiency with increased birnessite activity indicates inhibition by terminal acceptors and/or its toxicity which was also indicated by lactate oxidation results.

In terms of community structure, with increasing birnessite activities the relative abundance of fermentative Firmicutes decreased and Proteobacteria increased. From all the OTUs that were enriched, only the genus *Shewanella* contains well-studied metal reducing species (Hau and Gralnick, 2007; Zhong et al., 2018). Also, some representatives of the genera *Clostridium-sensu stricto* (Juan et al., 1999; Pham et al., 2003) have been described to perform extracellular electron transport and dissimilatory metal reduction. Other OTUs observed belong to either amino acid fermenting organisms such as *Proteiniclasticum* (Zhang et al., 2010), *Sedimentibacter* (Breitenstein et al., 2002) and VadinBC27- wastewater sludge group (Kim et al., 2018) or organic acids/sugar fermenting organisms such as the representatives of the family *Veillonellaceae* (Marchandin and Jumas-Bilak, 2014), genera *Citrobacter* (Kim et al., 2012), *Paludibacter* (Ueki et al., 2006), *Acidaminobacter* (Stams and Hansen, 1984), *Fusibacter* (Fadhlaoui et al., 2015), *Petrimonas* (Grabowski et al., 2005), and *Tyzzerella* (van der Wielen et al., 2002). *Veillonelaceae* is known to ferment lactate, with acetate and propionate as metabolic end products (Marchandin and Jumas-Bilak, 2014). *Telmatospirrilum* (Sizova et al., 2007), *Petrimonas* (Grabowski et al., 2005), *Paludibacter* (Ueki et al., 2006), *Acidaminobacter* (Stams and Hansen, 1984) and *Intestinimonas* (Kläring et al., 2013) are all lactate fermenting genera that produce acetate and propionate/formate. *Citrobacter* have been reported to utilize acetate as carbon source (Kim et al., 2012), which accumulated during lactate fermentation, therefore the increased *Citrobacter* relative sequence abundance with increased birnessite activity might be due to more acetate being available in the system for its growth.

An unexpected finding in our experiments was that fermenters and metal reducing bacteria such as *Shewanella* can compete for the same substrate. In traditional, canonical views of the anaerobic food chain, the fermenters would first ferment lactate providing substrates for metal reducing bacteria such as acetate and H_2_. This is not the case in our study where *Shewanella*, a known lactate oxidizer, competed with fermenters directly for the same substrate. The strict succession of redox processes has also been challenged by several other publication including Chen et al. (2017), Otte et al. (2018), and Kessler et al. (2019). Kessler et al. (2019) reported fermentation and respiration to be uncoupled processes, similarly to Otte et al. (2018) who found iron cycling to be uncoupled from geochemical gradients.

Recent research seems to confirm the possibility of fermentation and anaerobic respiration to co-occur as found in our study. Despite that, our systems do not reflect natural conditions, such as permanently anoxic sediments where carbon fermentation coupled with sulfate, Fe(III) and Mn(VI) reduction was previously reported (Canfield et al., 1993; Vandieken et al., 2014). In natural systems the birnessite concentrations would be much lower and community composition rather different. Hence, in permanently anoxic sediments mineralization of organic matter and anaerobic respiration would likely still be coupled.

## Conclusion

With increased birnessite activities a decrease in lactate consumption rate, altered propionate/acetate ratios and changes in microbial community were observed. The birnessite reduction efficiency decreased with increasing birnessite activities and was the highest at 10 mM. The relative sequence abundance of metal reducing bacteria did not equally increase with birnessite activities. The majority of OTUs enriched in our microcosms appeared to be involved in lactate/acetate fermentation (most Firmicutes and some Proteobacteria) or amino acid fermentation. Even at 40 mM of birnessite, metal reducing genus *Shewanella* had relative sequence abundance of only around 15%, indicating the importance of amino acid and organic acid fermenting microbes at this high birnessite activity. Increased production of acetate and CO_2_ with increasing birnessite activity indicated a shift from purely fermentative to partial oxidation to acetate coupled to dissimilatory metal reduction. Birnessite suppressed lactate fermentation, the faster form of lactate utilization. This enabled the metal reducing population to successfully compete for lactate, despite their slower conversion rate. It was also observed that the microbial community composition not only depended on the presence/absence of the electron acceptor, but also its activities, with richness and evenness of communities decreasing with increased birnessite activity.

## Author Contributions

BN conceived, planned, and executed the experiments, and wrote the manuscript with the input of co-authors. JZ helped in preparation of figures and uploaded the sequences to the SRA. SB provided editorial comments and access to analytical chemistry facilities. MS revised the manuscript.

## Conflict of Interest Statement

The authors declare that the research was conducted in the absence of any commercial or financial relationships that could be construed as a potential conflict of interest.

## References

[B1] Apprill A. Mcnally S. Parsons R. Weber L. (2015). Minor revision to V4 region SSU rRNA 806R gene primer greatly increases detection of SAR11 bacterioplankton. *Aquat. Microb. Ecol.* 75 129–137. 10.3354/ame01753

[B2] Breitenstein A. Wiegel J. Haertig C. Weiss N. Andreesen J. R. Lechner U. (2002). Reclassification of *Clostridium hydroxybenzoicum* as *Sedimentibacter hydroxybenzoicus* gen. nov., comb. nov., and description of *Sedimentibacter saalensis* sp. nov. *Int. J. Syst. Bacteriol.* 52(Pt 3), 801–807. 10.1099/ijs.0.01998-0 12054241

[B3] Bretschger O. Obraztsova A. Sturm C. A. In S. C. Gorby Y. A. Reed S. B. (2007). Current production and metal oxide reduction by *Shewanella oneidensis* MR-1 wild type and mutants. *Appl. Environ. Microbiol.* 73 7003–7012. 10.1128/AEM.01087-07 17644630 PMC2074945

[B4] Burdige D. J. Dhakar S. P. Nealson K. H. Burdige D. J. Dhakar S. P. Nealson K. H. (1992). Effects of manganese oxide mineralogy on microbial and chemical manganese reduction. *Geomicrobiol. J.* 10 27–48. 10.1080/01490459209377902

[B5] Burdige D. J. Nealson K. H. (1985). Chemical and microbiological studies of sulfide - mediated manganese reduction. *Geomicrobiol. J.* 4 361–387. 10.1080/01490458609385944

[B6] Canfield D. E. Thamdrup B. Hansen J. W. (1993). The anaerobic degradation of organic matter in danish coastal sediments: iron reduction, manganese reduction, and sulfate reduction. *Geochim. Cosmochim. Acta* 57 3867–3883. 10.1016/0016-7037(93)90340-3 11537734

[B7] Caporaso J. G. Lauber C. L. Walters W. A. Berg-lyons D. Lozupone C. A. Turnbaugh P. J. (2010). Global patterns of 16S rRNA diversity at a depth of millions of sequences per sample. *Proc. Natl. Acad. Sci. U.S.A.* 108 4516–4522. 10.1073/pnas.1000080107 20534432 PMC3063599

[B8] Chen J. Hanke A. Tegetmeyer H. E. Kattelmann I. Sharma R. Hamann E. (2017). Impacts of chemical gradients on microbial community structure. *ISME J.* 11 920–931. 10.1038/ismej.2016.175 28094795 PMC5363838

[B9] Chubar N. Avramut C. Visser T. (2015). Formation of manganese phosphate and manganese carbonate during long-term sorption of Mn2+ by viable *Shewanella putrefaciens*: effects of contact time and temperature. *Environ. Sci. Process. Impacts* 17 780–790. 10.1039/C4EM00634H 25707532

[B10] Cologgi D. L. Speers A. M. Bullard B. A. Kelly S. D. Reguera G. (2014). Enhanced uranium immobilization and reduction by *Geobacter sulfurreducens* biofilms. *Appl. Environ. Microbiol.* 80 6638–6646. 10.1128/AEM.02289-14 25128347 PMC4249037

[B11] Das A. P. Sukla L. B. Pradhan N. Nayak S. (2011). Manganese biomining: a review. *Bioresour. Technol.* 102 7381–7387. 10.1016/j.biortech.2011.05.018 21632238

[B12] Dong X. Kleiner M. Sharp C. E. Thorson E. Li C. Liu D. (2017). Fast and simple analysis of MiSeq amplicon sequencing data with MetaAmp. *Front. Chem.* 8:1461. 10.3389/fmicb.2017.01461 28824589 PMC5540949

[B13] Esther J. Sukla L. B. Pradhan N. Panda S. (2014). Fe(III) reduction strategies of dissimilatory iron reducing bacteria. *Korean J. Chem. Eng.* 32 1–14. 10.1007/s11814-014-0286-x 11976100

[B14] Fadhlaoui K. Ben Hania W. Postec A. Fauque G. Hamdi M. Ollivier B. (2015). *Fusibacter fontis* sp. nov., a sulfur-reducing, anaerobic bacterium isolated from a mesothermic Tunisian spring. *Int. J. Syst. Evol. Microbiol.* 65 3501–3506. 10.1099/ijsem.0.000445 26296995

[B15] Fischer T. B. Heaney P. J. Post E. (2018). Changes in the structure of birnessite during siderophore-promoted dissolution: a time-resolved synchrotron X-ray diffraction study. *Chem. Geol.* 476 46–58. 10.1016/j.chemgeo.2017.11.003

[B16] Gong Y. Werth C. J. He Y. Su Y. Zhang Y. Zhou X. (2018). Intracellular versus extracellular accumulation of hexavalent chromium reduction products by *Geobacter sulfurreducens* PCA. *Environ. Pollut.* 240 485–492. 10.1016/j.envpol.2018.04.046 29754098

[B17] Grabowski A. Tindall B. J. Bardin V. Blanchet D. Jeanthon C. (2005). *Petrimonas sulfuriphila* gen. nov., sp. nov., a mesophilic fermentative bacterium isolated from a biodegraded oil reservoir. *Int. J. Syst. Evol. Microbiol.* 55 1113–1121. 10.1099/ijs.0.63426-0 15879242

[B18] Hau H. H. Gralnick J. A. (2007). Ecology and biotechnology of the genus *Shewanella*. *Annu. Rev. Microbiol.* 61 237–258. 10.1146/annurev.micro.61.080706.093257 18035608

[B19] Horvath A. S. Garrick L. V. Moreau J. W. (2014). Manganese-reducing *Pseudomonas fluorescens*-group bacteria control arsenic mobility in gold mining-contaminated groundwater. *Environ. Earth Sci.* 71 4187–4198. 10.1007/s12665-013-2809-x

[B20] Hunt K. A. Flynn J. M. Naranjo B. Shikhare I. D. Gralnick J. A. (2010). Substrate-level phosphorylation is the primary source of energy conservation during anaerobic respiration of *Shewanella oneidensis* strain MR-1. *J. Bacteriol.* 192 3345–3351. 10.1128/JB.00090-10 20400539 PMC2897674

[B21] Jin Q. (2012). Energy conservation of anaerobic respiration. *Am. J. Sci.* 312 573–628. 10.2475/06.2012.01

[B22] Johnson J. E. Savalia P. Davis R. Kocar B. D. Webb S. M. Nealson K. H. (2016). Real-Time manganese phase dynamics during biological and abiotic manganese oxide reduction. *Environ. Sci. Technol.* 50 4248–4258. 10.1021/acs.est.5b04834.Real-Time 27018915 PMC5502836

[B23] Juan S. Dobbin P. S. Carter J. P. Garc C. Von Hobe M. Powell A. K. (1999). Dissimilatory Fe(III) reduction by *Clostridium beijerinckii* isolated from freshwater sediment using Fe(III) maltol enrichment. *FEMS Microbiol. Lett.* 176 131–138. 10.1016/s0378-1097(99)00229-3 10418140

[B24] Kessler A. J. Chen Y.-J. Waite D. W. Hutchinson T. Koh S. Popa M. E. (2019). Bacterial fermentation and respiration processes are uncoupled in permeable sediments. *Nat. Microbiol.* 1. 30858573 10.1038/s41564-019-0391-z

[B25] Kim E. Lee J. Han G. Hwang S. (2018). Comprehensive analysis of microbial communities in full-scale mesophilic and thermophilic anaerobic digesters treating food waste-recycling wastewater. *Bioresour. Technol.* 259 442–450. 10.1016/j.biortech.2018.03.079 29609168

[B26] Kim Y. Lee S. Park B. Son M. Jung Y. Yang S. (2012). Proteomic analysis on acetate metabolism in *Citrobacter* sp. BL-4. *Int. J. Biol. Sci.* 8 66–78. 10.7150/ijbs.8.66 22211106 PMC3248649

[B27] Kläring K. Hanske L. Bui N. Charrier C. Blaut M. Haller D. (2013). *Intestinimonas butyriciproducens* gen. nov., sp. nov., a butyrate-producing bacterium from the mouse intestine. *Int. J. Syst. Evol. Microbiol.* 63 4606–4612. 10.1099/ijs.0.051441-0 23918795

[B28] Kouzuma A. Hashimoto K. Watanabe K. (2012). Roles of siderophore in manganese-oxide reduction by *Shewanella oneidensis* MR-1. *FEMS Microbiol. Lett.* 326 91–98. 10.1111/j.1574-6968.2011.02444.x 22092340

[B29] Liu C. Gorby Y. A. Zachara J. M. Fredrickson J. K. Brown C. F. (2002). Reduction kinetics of Fe(III), Co(III), U(VI), Cr(VI), and Tc(VII) in cultures of dissimilatory metal-reducing bacteria. *Biotechnol. Bioeng.* 80 637–649. 10.1002/bit.10430 12378605

[B30] Lovley D. (2013). “Dissimilatory Fe(III)- and Mn(IV)-Reducing Prokaryotes,” in *The Prokaryotes*, eds DeLong E. F. Lory S. Stackebrandt E. Thompson F. (New York, NY: Springer), 387–309.

[B31] Lovley D. R. (1991). Dissimilatory Fe(III) and Mn(IV) reduction. *Microbiol. Rev.* 55 259–287. 10.1016/S0065-2911(04)49005-5 1886521 PMC372814

[B32] Luther G. W. I. (2007). Manganese(II) oxidation and Mn(IV) reduction in the environment — two one-electron transfer steps versus a single two-electron step. *Geomicrobiol. J.* 22 195–203. 10.1080/01490450590946022

[B33] Madigan M. T. Martinko J. M. Bender K. S. Buckley D. H. Stahl A. D. Brock T. (2017). “Catabolism of organic compounds,” in *Brock Biology of Microorganisms*, (Boston: Pearson), 372–411.

[B34] Marchandin H. Jumas-Bilak E. (2014). “The Family Veillonellaceae,” in *The Prokaryotes*, eds Rosenberg E. DeLong E. F. Lory S. Stackebrandt E. Thompson F. (Berlin: Springer), 433–453. 10.1007/978-3-642-30120-9_361

[B35] Marsili E. Baron D. B. Shikhare I. D. Coursolle D. Gralnick J. A. Bond D. R. (2008). *Shewanella* secretes flavins that mediate extracellular electron transfer. *Proc. Natl. Acad. Sci. U.S.A.* 105 3968–3973. 10.1073/pnas.0710525105 18316736 PMC2268775

[B36] McKenzie R. M. (1971). The synthesis of birnessite, cryptomelane, and some other oxides and hydroxides of manganese. *Mineral. Mag.* 38 493–502. 10.1180/minmag.1971.038.296.12

[B37] Methé B. A. Nelson K. E. Eisen J. A. Paulsen I. T. Nelson W. Heidelberg J. F. (2003). Genome of *Geobacter sulfurreducens*: metal reduction in subsurface environments. *Science* 302 1967–1969. 10.1126/science.1088727 14671304

[B38] Müller V. (2001). Bacterial fermentation. *Encycl. Life Sci.* (Chichester: John Wiley & Sons), 1–7.

[B39] Nevin K. P. Lovley D. R. (2000). Potential for nonenzymatic reduction of Fe(III) via electron shuttling in subsurface sediments. *Environ. Sci. Technol.* 34 2472–2478. 10.1021/es991181b

[B40] Novotnik B. Zuliani T. Ščančar J. Milačič R. (2014). Inhibition of the nitrification process in activated sludge by trivalent and hexavalent chromium, and partitioning of hexavalent chromium between sludge compartments. *Chemosphere* 105 87–94. 10.1016/j.chemosphere.2013.12.096 24462082

[B41] Otte J. M. Harter J. Laufer K. Blackwell N. Straub D. Kappler A. (2018). The distribution of active iron-cycling bacteria in marine and freshwater sediments is decoupled from geochemical gradients. *Environ. Microbiol.* 20 2483–2499. 10.1111/1462-2920.14260 29708639

[B42] Parada A. E. Needham D. M. Fuhrman J. A. (2016). Every base matters: assessing small subunit rRNA primers for marine microbiomes with mock communities, time series and global field samples. *Environ. Microbiol.* 18 1403–1414. 10.1111/1462-2920.13023 26271760

[B43] Parker N. Schneegurt M. Tu A.-H. T. Forster B. M. Lister P. (2016). *Fermentation, in Microbiology (OpenStax)*. Available at: https://courses.lumenlearning.com/microbiology/chapter/fermentation/. 10.1111/1462-2920.13023 (accessed July 5, 2018).

[B44] Pham C. A. Jung S. J. Phung N. T. Lee J. Chang I. S. Kim B. H. (2003). A novel electrochemically active and Fe(III)-reducing bacterium phylogenetically related to *Aeromonas hydrophila*, isolated from a microbial fuel cell. *FEMS Microbiol. Lett.* 223 129–134. 10.1016/S0378-1097(03)00354-359 12799011

[B45] Post J. E. Heaney P. J. Hanson J. (2002). Rietveld refinement of a triclinic structure for synthetic Na-birnessite using synchrotron powder diffraction data. *Powder Diffr.* 17 218–221. 10.1154/1.1498279

[B46] R Studio (2015). *RStudio: Integrated Development Environment for R*. Available at: http://www.rstudio.com/ (accessed January 3, 2018).

[B47] Richter K. Schicklberger M. Gescher J. (2012). Dissimilatory reduction of extracellular electron acceptors in anaerobic respiration. *Appl. Environ. Microbiol.* 78 913–921. 10.1128/AEM.06803-11 22179232 PMC3273014

[B48] Roden E. E. Kappler A. Bauer I. Jiang J. Paul A. Stoesser R. (2010). Extracellular electron transfer through microbial reduction of solid-phase humic substances. *Nat. Geosci.* 3 417–421. 10.1038/ngeo870

[B49] Seeliger S. Janssen P. H. Schink B. (2002). Energetics and kinetics of lactate fermentation to acetate and propionate via methylmalonyl-CoA or acrylyl-CoA. *FEMS Microbiol. Lett.* 211 65–70. 10.1016/s0378-1097(02)00651-1 12052552

[B50] Sharp C. E. Urschel S. Dong X. Brady A. L. Slater G. F. Strous M. (2017). Robust, high-productivity phototrophic carbon capture at high pH and alkalinity using natural microbial communities. *Biotechnol. Biofuels* 10:84. 10.1186/s13068-017-0769-1 28367229 PMC5372337

[B51] Shi L. Squier T. C. Zachara J. M. Fredrickson J. K. (2007). Respiration of metal (hydr)oxides by *Shewanella* and *Geobacter*: a key role for multihaem c-type cytochromes. *Mol. Microbiol.* 65 12–20. 10.1111/j.1365-2958.2007.05783.x 17581116 PMC1974784

[B52] Sizova M. V. Panikov N. S. Spiridonova E. M. Slobodova N. V. Tourova T. P. (2007). Novel facultative anaerobic acidotolerant *Telmatospirillum siberiense* gen. nov. sp. nov. isolated from mesotrophic fen. *Syst. Appl. Microbiol.* 30 213–220. 10.1016/j.syapm.2006.06.003 16876366

[B53] Stams A. J. M. Hansen T. A. (1984). Fermentation of glutamate and other compounds by *Acidaminobacter hydrogenoformans* gen. nov. sp. nov., an obligate anaerobe isolated from black mud. Studies with pure cultures and mixed cultures with sulfate-reducing and methanogenic bacteria. *Arch. Microbiol.* 137 329–337. 10.1007/BF00410730

[B54] Straub K. L. Schink B. (2004). Ferrihydrite-dependent growth of *Sulfurospirillum deleyianum* through electron transfer via sulfur cycling. *Appl. Environ. Microbiol.* 70 5744–5749. 10.1128/AEM.70.10.5744 15466509 PMC522073

[B55] Tessier A. Campbell P. G. C. Bisson M. (1979). Sequential extraction procedure for the speciation of particulate trace metals. *Anal. Chem.* 51 844–851. 10.1021/ac50043a017

[B56] Ueki A. Akasaka H. Suzuki D. Ueki K. (2006). *Paludibacter propionicigenes* gen. nov., sp. nov., a novel strictly anaerobic, gram-negative, propionate-producing bacterium isolated from plant residue in irrigated rice-field soil in Japan. *Int. J. Syst. Evol. Microbiol.* 56(Pt 1), 39–44. 10.1099/ijs.0.63896-0 16403864

[B57] van der Wielen P. W. J. J. Rovers G. M. L. L. Scheepens J. M. A. Biesterveld S. (2002). *Clostridium lactatifermentans* sp. nov., a lactate-fermenting anaerobe isolated from the caeca. *Int. J. Syst. Evol. Microbiol.* 52 921–925. 10.1099/ijs.0.02048-0 12054258

[B58] Vandieken V. Finke N. Thamdrup B. (2014). Hydrogen, acetate, and lactate as electron donors for microbial manganese reduction in a manganese-rich coastal marine sediment. *FEMS Microbiol. Ecol.* 87 733–745. 10.1111/1574-6941.12259 24266405

[B59] Von Canstein H. Ogawa J. Shimizu S. Lloyd J. R. (2008). Secretion of flavins by *Shewanella species* and their role in extracellular electron transfer. *Appl. Environ. Microbiol.* 74 615–623. 10.1128/AEM.01387-07 18065612 PMC2227709

[B60] Wang Z. Lee S. W. Kapoor P. Tebo B. M. Giammar D. E. (2013). Uraninite oxidation and dissolution induced by manganese oxide: a redox reaction between two insoluble minerals. *Geochim. Cosmochim. Acta* 100 24–40. 10.1016/j.gca.2012.09.053

[B61] Ying S. C. Kocar B. D. Gri S. D. Fendorf S. (2011). Competitive microbially and Mn oxide mediated redox processes controlling arsenic speciation and partitioning. *Environ. Sci. Technol.* 45 5572–5579. 10.1021/es200351m 21648436

[B62] Zhang H. Li Y. Wang X. Lu A. Ding H. Zeng C. (2015). Aerobic and anaerobic reduction of birnessite by a novel *Dietzia* strain. *Geochem. Trans.* 16 7–11. 10.1186/s12932-015-0026-0 26257581 PMC4528715

[B63] Zhang P. Shen Y. Guo J. S. Li C. Wang H. Chen Y.-P. (2015). Extracellular protein analysis of activated sludge and their functions in wastewater treatment plant by shotgun proteomics. *Sci. Rep.* 5:12041. 10.1038/srep12041 26160685 PMC4498230

[B64] Zhang K. Song L. Dong X. (2010). *Proteiniclasticum ruminis* gen. nov., sp. nov., a strictly anaerobic proteolytic bacterium isolated from yak rumen. *Int. J. Syst. Evol. Microbiol.* 60 2221–2225. 10.1099/ijs.0.011759-0 19915115

[B65] Zhong C. Han M. Yu S. Yang P. Li H. Ning K. (2018). Pan-genome analyses of 24 *Shewanella* strains re-emphasize the diversification of their functions yet evolutionary dynamics of metal-reducing pathway. *Biotechnol. Biofuels* 11:193. 10.1186/s13068-018-1201-1 30026808 PMC6048853

